# Primary tuberculosis of the thyroid gland complicated with abscess: Case report

**DOI:** 10.1016/j.idcr.2026.e02498

**Published:** 2026-01-19

**Authors:** Wondwosen Mengist Dereje, Desalegn Kefale Aegash, Alem Demissie Bogale, Samuel Addisu Abera, Mengist Asmamaw Tegegne, Abel Girma Demessie

**Affiliations:** a University of Gondar, College of Medicine and Health sciences, Department of Neurology, Gondar, Ethiopia; b University of Gondar, College of Medicine and Health sciences, Department of Surgery, Gondar, Ethiopia; c University of Gondar, College of Medicine and Health sciences, Department of Pathology, Gondar, Ethiopia

**Keywords:** Thyroid gland, Tuberculosis, Acid fast bacilli, Thyroidectomy, Anti-tuberculosis medication, Case report

## Abstract

**Introduction and importance:**

Tuberculosis (TB), caused by *Mycobacterium tuberculosis*, mainly affects the lungs and lymph nodes but rarely involves the thyroid. Thyroid TB is usually secondary to infection elsewhere, while primary thyroid TB, where the thyroid is the initial site, is extremely rare and more common in patients with preexisting thyroid disease. Its nonspecific presentation often delays diagnosis and treatment. This case highlights the need for a high index of suspicion for early detection and management.

**Case presentation:**

A 40-year-old female farmer presented with a 20-year history of anterior neck swelling, which increased in size over two months. She developed pain, bilateral cervical lymphadenopathy, and low-grade fever. Examination revealed a sinus tract with purulent discharge from the right thyroid lobe and multiple cervical lymph nodes. Ultrasound suggested thyroid TB; CT scan suggested thyroid cancer with nodal metastasis. Fine needle aspiration cytology confirmed TB. Right thyroid lobectomy with left subtotal thyroidectomy was performed, and histopathology confirmed thyroid tuberculosis. She was treated with first-line anti-TB medications and followed for six months, showing full improvement.

**Conclusion:**

Primary thyroid TB is exceptionally rare and can mimic malignancy. FNAC and biopsy can confirm TB by demonstrating granulomatous inflammation with caseation and identifying *Mycobacterium tuberculosis*. Clinicians should consider TB in thyroid lesions, particularly in endemic areas, to ensure timely diagnosis and appropriate treatment.

## Highlights

• Tuberculosis (TB) is a contagious infection affecting lungs and lymph nodes; thyroid involvement is rare.
• Primary thyroid TB is an unusual presentation, often leading to delayed diagnosis and treatment.
• Thyroid TB is often mistaken for cancer or other disorders; definitive diagnosis needs FNAC or biopsy.
• Early recognition and prompt management are critical to improving outcomes in such atypical cases.

## Background

Thyroid tuberculosis (TT) is an exceptionally rare clinical entity. The first documented case was reported by Lebert in 1862, in a young woman who succumbed to miliary tuberculosis; autopsy revealed tuberculosis involvement of the thyroid gland [1].

Since then, most confirmed cases of thyroid tuberculosis have been diagnosed either postoperatively, following thyroidectomy for suspected neoplasm or abscess, or incidentally at autopsy [2], [3], [4]. When tuberculosis does involve the thyroid gland, it is typically secondary to disseminated (miliary) disease rather than a primary focus, which underscores the unusual nature of our case. The rarity of primary thyroid tuberculosis highlights the importance of maintaining a high index of suspicion, particularly in endemic regions, to ensure timely diagnosis and appropriate management.

## Case presentation

A 40-year-old female patient presented with a progressively enlarging anterior neck mass, which she had noticed for the past 20 years. Over the last two months, the mass had grown rapidly, and in the past three weeks, she began experiencing localized pain. Concurrently, she also developed bilateral posterior neck swellings and reported low-grade, intermittent fever of the same three-week duration but she denied drenching night sweat and weight loss.

She also denied experiencing cough, difficulty swallowing (dysphagia), or shortness of breath. Additionally, she reported no symptoms of hyper- or hypothyroidism, including palpitations, menstrual irregularities, or intolerance to heat or cold.

On initial assessment, she appeared clinically stable and comfortable. Her vital signs were within normal limits: blood pressure of 110/70 mmHg, pulse rate of 82 beats per minute, respiratory rate of 19 breaths per minute, and a body temperature of 36.9°C. Her body mass index (BMI) was 19 kg/m².

On physical examination, a 7 × 6 cm tender anterior neck mass was observed, more prominent on the right side. A sinus tract was noted over the right thyroid lobe, with active purulent discharge. Multiple non-tender bilateral cervical lymph nodes were also palpable. No other superficial lymphadenopathy was detected.

An initial clinical diagnosis of superinfected thyroid malignancy with cervical lymph node metastasis was made. Laboratory investigations were conducted, revealing the following: Complete Blood Count (CBC)**:** WBC 6.5 × 10 ³ /µL, Neutrophils 56 %, Lymphocytes 33 %, Hemoglobin 13.5 g/dL, Hematocrit 38.8 %, Platelet count 406 × 10 ³ /µL. Thyroid Function Tests: TSH 4.7 µIU/mL (reference: 0.3–4.5), Free T3 3.11 pg/mL (reference: 2–4.2), Free T4 1.43 ng/dL (reference: 0.9–1.75). Liver Function Tests**:** ALP 53 U/L, SGOT (AST) 14 U/L, SGPT (ALT) 14 U/L. Renal Function Tests**:** BUN 16 mg/dL, Serum Creatinine 0.46 mg/dL. Provider-Initiated Testing and Counseling was done and it was non-reactive**.**

Inflammatory markers, such as CRP and ESR, were not performed because they were unavailable in our setting, despite their clinical importance.

**Fig. 1:**
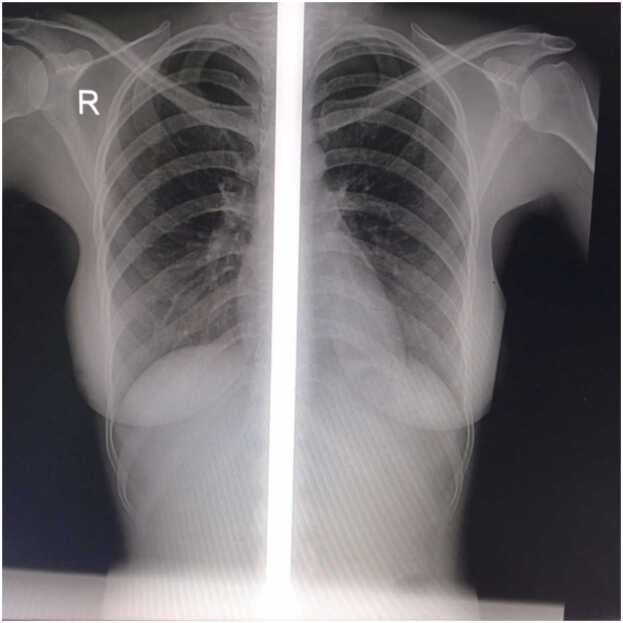
Normal chest radiography of the patient.

ECG and echocardiography**:** Normal findings. Chest X-ray**:** No abnormalities detected (Fig. 1).

Neck ultrasound showed enlarged thyroid gland with cystic mass and debrinous thick fluid collection and visible sinus tract on right lobe. It were also showed enlarged, hypoechoic cervical lymph nodes suggesting disseminated tuberculosis involving the lymph nodes and thyroid gland with abscess collection, and recommended further evaluation with Fine Needle Aspiration Cytology (FNAC). However, a contrast-enhanced CT scan of the neck revealed an enlarged right thyroid lobe containing a heterogeneous pre-contrast mass with coarse calcifications and central necrosis. The mass demonstrated heterogeneous post-contrast enhancement and extended into the prethyroid tissues. It also caused a mass effect, displacing the common carotid arteries. Additionally, cervical lymphadenopathy with homogeneous enhancement was noted. These findings are suggestive of thyroid carcinoma with cervical lymph node metastasis.

Given the long-standing nature of the thyroid mass, our initial consideration was a benign multinodular goiter. However, since thyroid tuberculosis is more likely to occur in glands with preexisting thyroid disease, and based on the clinical and cytological findings, we considered thyroid tuberculosis as top differential diagnosis.

**Fig. 2:**
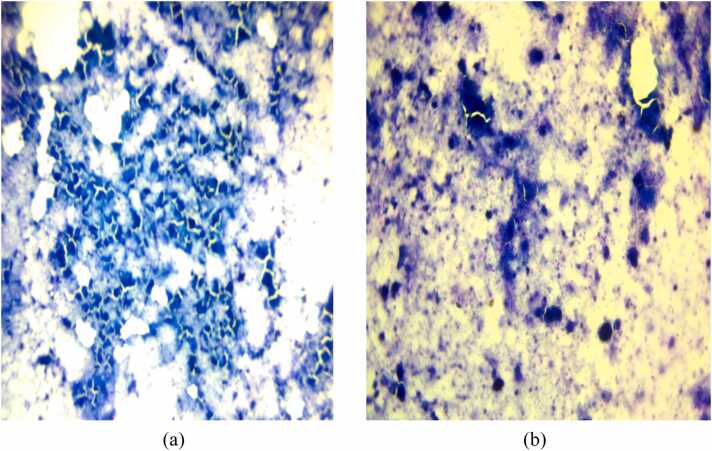
(A-B): FNAC findings, prominent necrosis with some granulomatous changes suggestive of tuberculosis.

In light of the suspected infection, the patient was started empirically on intravenous Ceftriaxone 1 g twice daily. FNAC of the thyroid lesion was performed, revealing necrotizing granulomatous inflammation consistent with tuberculosis (Fig. 2), and a tissue biopsy was recommended to confirm the diagnosis.

The findings were discussed with the patient and her family, and surgical management was decided for diagnosis as well as management of abscess collection. After obtaining written informed consent, the patient was taken to the operating room.

She subsequently underwent a right thyroid lobectomy with left subtotal thyroidectomy. Following the procedure, the patient was transferred to the post-anesthesia care unit, where she remained for 8 h, and was then moved to the recovery unit for the next 24 h. She was later transferred to the ward, where she received wound care and follow-up, and was discharged on the fourth postoperative day.

**Fig. 3:**
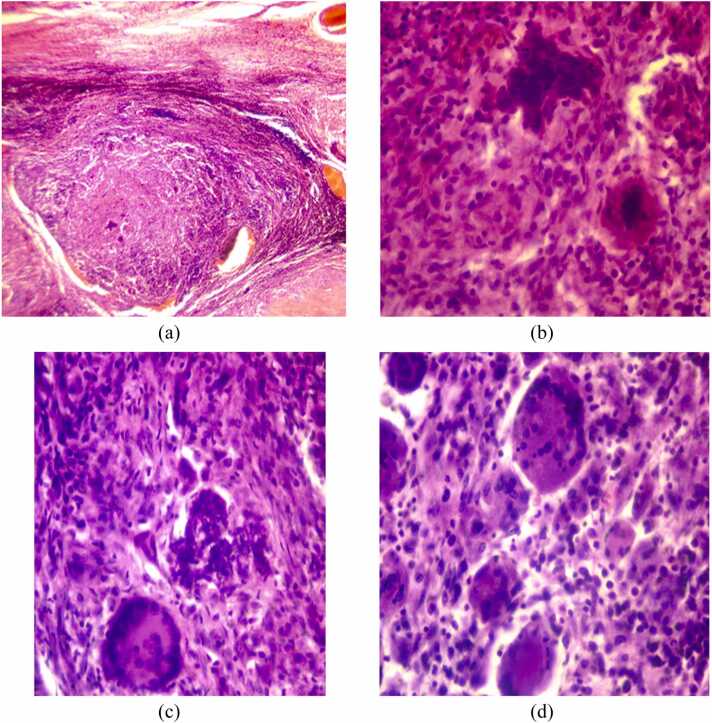
(A-D): Histopathologic images from the biopsy sample showing granulomatous changes with caseous central necrosis.

The resected tissue was sent for histopathological examination, which confirmed the diagnosis of tuberculosis (Fig. 3).

Following surgical management, the patient was started on anti-tuberculosis therapy and was followed up at our outpatient clinic. Her follow-up visits at one week, two weeks, four weeks, and then monthly until completion of anti-tuberculosis treatment revealed no new complaints, with complete resolution of her previous symptoms, including neck pain, fever, and neck mass.

Thyroid function tests were performed during her follow-up visits, and all results were within normal limits throughout her records.

After six months of treatment with anti-tuberculosis medication (with a two-month intensive phase (Isoniazid + Rifampicin + Pyrazinamide + Ethambutol) followed by a four-month continuation phase (Isoniazid + Rifampicin), all her symptoms resolved and finally discharged from our hospital.

## Discussion

Tuberculosis of the thyroid gland is an exceedingly rare condition, and its true incidence remains uncertain [5].

Reported estimates suggest an incidence ranging between 0.1 % and 0.4 % in clinical and autopsy series [6], [7], [8].

This rarity is thought to reflect the thyroid gland’s inherent resistance to infection, which can be attributed to several protective factors. These include its rich lymphatic and vascular supply, which facilitates immune surveillance; a well-developed fibrous capsule that acts as a barrier; the high iodine content of the gland; and the bactericidal properties of both iodine and thyroid colloid [3], [7], [9], [10], [11].

Collectively, these features create a local environment that is hostile to Mycobacterium tuberculosis, helping to explain the gland’s relative immunity to tuberculous involvement.

Tuberculosis of the thyroid gland may present as a primary infection within the gland itself or, more commonly, as a secondary manifestation of tuberculous disease elsewhere in the body. The most frequent pattern of thyroid involvement is through miliary dissemination, in which the gland becomes one of several organs seeded during widespread hematogenous spread. Studies have shown that approximately 7 % of patients with miliary tuberculosis exhibit thyroid involvement as part of the disseminated disease process, typically manifesting as a goiter without significant impairment of thyroid function [12].

A less common form is focal or caseous tuberculosis of the thyroid, which may present clinically as a thyroid nodule, an abscess, or a diffuse goiter [12]. Recognizing these varied presentations is crucial for timely diagnosis and appropriate management, given the gland’s rare susceptibility to tuberculous infection.

Secondary involvement of the thyroid gland is most commonly seen as a result of hematogenous dissemination or by direct extension from an active tuberculous focus in adjacent structures, such as the larynx or cervical lymph nodes [3], [10], [13].

Due to its rarity, tuberculosis of the thyroid gland is often misdiagnosed as thyroid cancer [14], subacute thyroiditis [15], or acute bacterial (staphylococcal) thyroid infection [16].

The most common symptoms of thyroid tuberculosis include fatigue, fever, night sweats, and weight loss. Pressure-related symptoms such as dysphagia, dyspnea, and dysphonia may also occur due to gland enlargement [3], [4], [7], [17].

Laboratory findings often reveal elevated erythrocyte sedimentation rate (ESR) and C-reactive protein (CRP) levels [18].

In the reported case, erythrocyte sedimentation rate (ESR) and C-reactive protein (CRP) measurements could not be obtained because these tests were unavailable in our setting at the time of evaluation.

Thyroid function tests are usually normal, although cases of thyrotoxicosis or myxedema have occasionally been reported [3], [19].

The diagnosis is confirmed through histological examination, which demonstrates multiple coalescing and caseating epithelioid cell granulomas along with multinucleated giant cells [6], [20].

Tuberculosis of the thyroid can present in various histopathological forms, including multiple thyroid granulomas, goiter with caseation, cold abscess, chronic fibrosing thyroiditis, and acute abscess [6], [7], [8].

Fine needle aspiration biopsy (FNAB) is a simple, rapid, cost-effective, and widely used diagnostic technique for evaluating thyroid lesions. In endemic regions, the diagnostic yield of thyroid tuberculosis using FNAB has been reported to range from 0.6 % to 1.15 % among all thyroid lesions. However, despite its utility, FNAB does not entirely eliminate the need for surgical intervention in certain cases [3].

Historically, the diagnostic criteria for thyroid tuberculosis, described as early as 1939, included the demonstration of acid-fast bacilli (AFB) within thyroid tissue, evidence of a necrotic or abscessed gland, and identification of tuberculous foci in other sites [20].

In contemporary practice, however, it is recognized that AFB are not consistently detected on smears. Therefore, the presence of multiple coalescing and caseating epithelioid cell granulomas, accompanied by Langhans giant cells and peripheral lymphocytic infiltration, is now considered diagnostic of thyroid tuberculosis even in the absence of demonstrable bacilli [11], [20].

In addition to histopathological examination, demonstrating *Mycobacterium tuberculosis* through bacterial culture provides a definitive diagnosis of the disease. However, a negative culture result does not completely exclude tuberculosis, as up to 50 % of cultures from extrapulmonary specimens may yield false negatives due to the paucibacillary nature of the infection [21].

Imaging techniques are generally not very helpful in establishing the diagnosis of tuberculous thyroiditis [22].

Ultrasonography typically demonstrates a heterogeneous, hypoechoic mass that may mimic a neoplastic lesion. An abscess appears anechoic and may show internal echoes [23].

Computed tomography (CT) can demonstrate a peripherally enhancing, low-density abscess associated with regional lymphadenopathy [24].

Contrast-enhanced CT may aid in localizing the caseous necrotic lesion, which is characterized by a necrotic center with peripheral rim enhancement resulting from acute inflammation and thickening of adjacent tissues (the “dermal sign”) [22].

A recent study has described the MRI features of thyroid tuberculosis [25]. The normal thyroid gland appears homogeneously hyperintense relative to the neck muscles on both T1- and T2-weighted images. In contrast, tuberculous involvement of the thyroid demonstrates intermediate signal intensity due to the presence of densely cellular inflammatory granulation tissue, consisting of tuberculous granulomas with or without minimal necrosis [25].

However, this appearance is nonspecific, as thyroid carcinoma may show similar imaging features. A subcutaneous abscess typically appears hypointense on T1-weighted images and hyperintense on T2-weighted images and may demonstrate peripheral rim enhancement on contrast-enhanced MRI [25].

PCR is a modern diagnostic tool that supports diagnosis and limits the requirement for invasive procedures [26].

However, in the reported case, PCR could not be performed because a PCR machine was not available at the hospital.

The primary treatment modalities for thyroid tuberculosis include antituberculosis therapy combined with surgical removal of the affected thyroid tissue. Another approach involves repeated drainage of abscesses through needle aspiration, used alongside antituberculosis medication [20], [27].

More recently, some studies have suggested that antituberculous drugs alone may be sufficient to achieve complete resolution in selected cases [11].

The standard antituberculosis regimen usually consists of a six-month course, with a two-month intensive phase (Isoniazid + Rifampicin + Pyrazinamide + Ethambutol) followed by a four-month continuation phase (Isoniazid + Rifampicin) [18], [20], [21].

Patients respond well to surgical intervention combined with antituberculous therapy [28], [29].

In the reported case, the need for surgical intervention was supported by the presence of an abscess collection, both clinically and on ultrasonographic evaluation. Furthermore, FNAC was nondiagnostic, making biopsy necessary. Given the diagnostic dilemma and the presence of an abscess, surgical management was considered appropriate.

Following the surgery, our patient was treated with the standard antituberculosis regimen for six months and get improved.

## Conclusion

Tuberculosis (TB), caused by *Mycobacterium tuberculosis* (MT), is a highly contagious infectious disease that can affect nearly any organ in the body. However, involvement of the thyroid gland is extremely rare. When the thyroid is affected, it is typically due to hematogenous dissemination from a primary focus such as the lungs or lymph nodes. Primary thyroid tuberculosis is an exceptionally uncommon occurrence in clinical practice. Because of its rarity, physicians often consider more common differential diagnoses, such as thyroid cancer, when evaluating thyroid lesions. A high index of suspicion is essential for the timely diagnosis of such rare cases, allowing for early initiation of appropriate treatment and improved patient outcomes before complications arise.

## Lists of abbreviations

TB Tuberculosis

FNAC fine needle aspiration cytology

CBC complete blood count

WBC White blood cells

AFB Acid-fast bacilli

TSH Thyroid stimulating hormone

CT scan Computed tomography scan

## Methods

The study has been reported in line with SCARE criteria.

## CRediT authorship contribution statement

**Desalegn Kefale Aegash:** Writing – review & editing, Writing – original draft, Visualization, Validation, Investigation. **Alem Demissie Bogale:** Writing – review & editing, Writing – original draft, Investigation. **Wondwosen Mengist Dereje:** Writing – review & editing, Writing – original draft, Visualization, Validation, Formal analysis, Data curation, Conceptualization. **Samuel Addisu Abera:** Writing – review & editing, Writing – original draft, Investigation. **Mengist Asmamaw Tegegne:** Writing – review & editing, Writing – original draft, Investigation. **Abel Girma Demessie:** Writing – review & editing, Writing – original draft, Supervision, Investigation.

## Consent

Written informed consent was obtained from the patient's parents for publication and any accompanying images. A copy of the written consent is available for review by the Editor-in-Chief of this journal on request.

## Ethical approval

Ethical approval for this study was provided by the Ethical Committee of our institution at Gondar university hospital, Gondar, Ethiopia on August, 2025.

## Funding

No funding.

## Declaration of Competing Interest

The authors declare that there are no known financial conflicts of interest or personal relationships that could have influenced, or appear to have influenced, the work presented in this case report. All authors have contributed independently and objectively, and there are no affiliations or involvements with any organization or entity with a financial or non-financial interest in the subject matter discussed in this report.

## Data Availability

Due to privacy of the patient we decline to share data.

## References

[1] Goldfarb H., Schifrin D., Graig F.A. (1965 May). Thyroiditis caused by tuberculous abscess of the thyroid gland. CASE Rep Rev Lit Am J Med.

[2] Akbulut S., Gomceli I., Cakabay B., Arikok A.T., Sezgin A., Bakir S. (2010 Feb 1). Clinical presentation of primary thyroid tuberculosis. Thyroid.

[3] Bulbuloglu E., Ciralik H., Okur E., Ozdemir G., Ezberci F., Cetinkaya A. (2006). Tuberculosis of the thyroid gland: review of the literature. World J Surg.

[4] Razmpa A., Sharifian H., Sadeghi Hasanabadi M., Ilami A., Shahinfar S.H. (2007). Clinical and paraclincial aspect of thyroid tuberculosis. Acta Med Iran.

[5] Bolis G.B. (1970). Tuberculosi fibrosa della ghiandola tiroide. Lav Ist Anat Istol Patol Univ Perugia.

[6] Girgin S., Gedik E., Büyükbayram H. (2007). Asymptomatic thyroid tuberculosis in a multinodular goitre patient: a case report. Acta Chir Belg.

[7] Bilgin G., Hasanoğlu A., Çakır B., Tumer H., Ustun H., Kusdemir A. (2005). Tuberculosis of thyroid with cord paralysis: A report of a case. Med J Kocatepe.

[8] Ghosh A., Saha S., Bhattacharya B., Chattopadhay S. (2007). Primary tuberculosis of thyroid gland: a rare case report. Am J Otolaryngol Head Neck Med Surg.

[9] Dawka S., Jayakumar J., Ghosh A. (2007). Primary tuberculosis of the thyroid gland. Kathmandu Univ Med J.

[10] Mpikashe P., Sathekge M.M., Mokgoro N.P. (2004). Tuberculosis of the thyroid gland: a case report. SA Fam Pr.

[11] Terzidis K., Tourli P., Kiapekou E., Alevizaki M. (2007). Thyroid tuberculosis. Hormones.

[12] Hizawa K., Okamura K., Sato K., Kuroda T., Yoshinari M., Ike-noue H. (1990). Tuberculous thyroiditis and mil-iary tuberculosis manifested postpartum in a patient withthyroid carcinoma. Endocrinol Jpn.

[13] Sharma A.B., Kumar L.D., Sharma H.D., Debnath K., Naorem S. (2006). Primary tuberculosis of the thyroid gland – a rarity. J Indian Acad Clin Med.

[14] Allan R., O’Flynn W., Clarke S.E.M. (1990). Tuberculosis of thethyroid bed presenting as recurrent medullary thyroid car-cinoma. Tubercle.

[15] Sachs M.K., Dickenson G., Amazon K. (1988). Tuberculous adeni-tis of the thyroid mimicking subacute thyroiditis. Am J Med85.

[16] Cheah O.S.H. (1987). Tuberculosis of the thyroid gland: a case report. Med J Malays.

[17] Pandit A.A., Joshi A.S., Ogale S.B., Sheode J.H. (1997). Tuberculosis of thyroid gland. Ind J Tub.

[18] Fica S., Barbu C., Sirbu A., Terzea D., Clatici V., Loachim D. (2005). Rare form of tuberculosis presented as thyroid mass. Acta Endocrinol.

[19] Hashemi S.H., Nadi E. (2006). Thyroid tuberculosis presenting as a cystic nodule. Iran J Med Sci.

[20] Andrius Simkus (2004). Thyroid tuberculosis. Medicina.

[21] Jacobs R.F., Starke J.R. (1993). Tuberculosis in children. Med Clin North Am.

[22] Kang B.C., Lee S.W., Shim S.S., Choi H.Y., Baek S.Y., Cheon Y.J. (2000). US and CT findings of tuberculosis of the thyroid: three case reports. Clin Imaging.

[23] Parmar H., Hashmi M., Rajput A., Patankar T., Castillo M. (2002). Acute tuberculous abscess of the thyroid gland. Australas Radiol.

[24] Satish K.G., Bandhopadhya P.K., Garg K., Dash R.J. (1986). Tuberculosis of the thyroid gland. Lung India.

[25] Madhusudhan K.S., Seith A., Khadgawat R., Das P., Mathur S. (2009). Tuberculosis of the thyroid gland: magnetic resonance imaging appearances. Singap Med J.

[26] Bansal L.K., Gupta S., Gupta A.K., Chaudhary P. (2021 Apr 1). Thyroid tuberculosis. Indian J Tuberc.

[27] Safarpor F., Hedayeti Omami M.H., Mohammadi F., Hoda S., Safarpor D. (2007). Thyroid tuberculosis. Iran Red Crescent Med J.

[28] Chung S.Y., Oh K.K., Chang H.S. (2002). Sonographic findings of tuberculous thyroiditis in a patient with Behçet's syndrome. J Clin Ultrasound.

[29] Tabacu E., Galie N., Galbenu P., Mitrea M. (2000). Thyroid tuberculosis--a clinical case. Pneumologia.

